# General Pathways of Pain Sensation and the Major Neurotransmitters Involved in Pain Regulation

**DOI:** 10.3390/ijms19082164

**Published:** 2018-07-24

**Authors:** Mun Fei Yam, Yean Chun Loh, Chu Shan Tan, Siti Khadijah Adam, Nizar Abdul Manan, Rusliza Basir

**Affiliations:** 1 Department of Human Anatomy, Faculty of Medicine and Health Sciences, Universiti Putra Malaysia, Serdang 43400, Malaysia; sk.adam@upm.edu.my (S.K.A.); nizar@upm.edu.my (N.A.M.); 2 Department of Pharmacology, School of Pharmaceutical Sciences, Universiti Sains Malaysia, Minden 11800, Malaysia; lyc14_pha052@student.usm.my (Y.C.L.); chushan@usm.my (C.S.T.)

**Keywords:** pain sensitization, neurotransmitters, nociceptive, inflammatory, neuropathic, presynaptic and postsynaptic, pain transmission, neurons, synaptic transmission

## Abstract

Pain has been considered as a concept of sensation that we feel as a reaction to the stimulus of our surrounding, putting us in harm’s way and acting as a form of defense mechanism that our body has permanently installed into its system. However, pain leads to a huge chunk of finances within the healthcare system with continuous rehabilitation of patients with adverse pain sensations, which might reduce not only their quality of life but also their productivity at work setting back the pace of our economy. It may not look like a huge deal but factor in pain as an issue for majority of us, it becomes an economical burden. Although pain has been researched into and understood by numerous researches, from its definition, mechanism of action to its inhibition in hopes of finding an absolute solution for victims of pain, the pathways of pain sensation, neurotransmitters involved in producing such a sensation are not comprehensively reviewed. Therefore, this review article aims to put in place a thorough understanding of major pain conditions that we experience—nociceptive, inflammatory and physiologically dysfunction, such as neuropathic pain and its modulation and feedback systems. Moreover, the complete mechanism of conduction is compiled within this article, elucidating understandings from various researches and breakthroughs.

## 1. Introduction

Pain is considered to be a human primate instinct and can be defined as a distressing sensation, as well as an emotional experience that is linked to actual or potential tissue damage, with the sole purpose of notifying the body’s defence mechanism to react towards a stimulus in order to avoid further tissue damages. The sensation of pain is associated with the activation of the receptors in the primary afferent fibers, which is inclusive of the unmyelinated C-fiber and myelinated Aσ-fiber. Both nociceptors remain silent during homeostasis in the absence of pain and are activated when there is a potential of noxious stimulus. The perception of a series of sensory events is required for the brain in order to detect pain and produce a response towards the threat. There are generally three main stages in the perception of pain. The first stage is pain sensitivity, followed by the second stage where the signals are transmitted from the periphery to the dorsal horn (DH), which is located in the spinal cord via the peripheral nervous system (PNS). Lastly, the third stage is to perform the transmission of the signals to the higher brain via the central nervous system (CNS). Typically, there are two routes for signal transmissions to be conducted: ascending and descending pathways. The pathway that goes upward carrying sensory information from the body via the spinal cord towards the brain is defined as the ascending pathway, whereas the nerves that goes downward from the brain to the reflex organs via the spinal cord is known as the descending pathway.

Pain is a vast subject and affects so many regions of an individual’s body that we feel pain originating from numerous roots, including cancer, fibromyalgia, neuropathic pain, persistent post-surgical pain, arthritis, childhood and adolescent pain, headache and migraine, orofacial pain, visceral pain, musculoskeletal pain and pelvic pain. According to the International Association for the Study of Pain (IASP), pain can be classified, based on the region of the body involved (e.g., head, visceral), pattern of occurrence’s duration (acute and chronic), or the system of which dysfunction that may cause the pain (e.g., gastrointestinal, nervous). However, it is suggested for pain to be classified based on only three characteristics: symptoms, mechanisms and syndromes. Thus, internationally pain has been classified into three major classes—nociceptive pain, neuropathic pain and inflammatory pain [1]. Primarily, both the CNS and PNS are involved in the mechanism and pathways of all variations of pain perception. The PNS comprises nerves and ganglia that are located outside the brain and spinal cord, mainly functioning to connect the CNS to organs and limbs in our body. On the other hand, the CNS is composed of the spinal cord and the brain, which is mainly responsible for integrating and intepreting the information sent from the PNS, and subsequently coordinating all the activities in our bodies, before sending response towards the effector organs.

## 2. Basic Mechanisms of Pain

**Figure 1:**
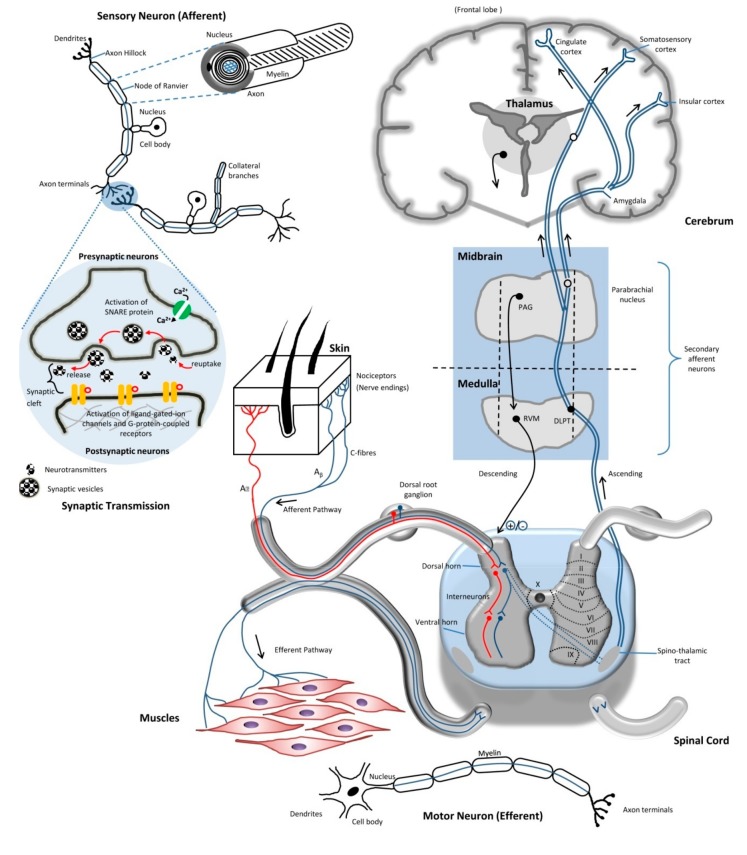
The basic route of pain transmission upon noxious stimuli in ascending and descending order, and the illustration of synaptic transmission in synaptic cleft.

Fundamentally, the basic pain mechanism undergoes three events—transduction, transmission and modulation when there is a presence of noxious stimuli. For instance, transduction occurs along the nociceptive pathway following such order: (1) stimulus events are converted to chemical tissue events; (2) chemical tissue and synaptic cleft events are then changed into electrical events in the neurons; and (3) electrical events in the neurons are transduced as chemical events at the synapses. After the completion of transduction, the following mechanism would be transmission. It takes place by transmitting the electrical events along the neuronal pathways, while neurotransmitters in the synaptic cleft transmit information from a post-synaptic terminal of one cell to a pre-synaptic terminal of another. Meanwhile, the modulation event takes place at all level of nociceptive pathways through the primary afferent neuron, DH and higher brain center by up- or down-regulation. All these lead to one end result, and the pathway of pain has been initiated and completed, thus allowing us to feel the painful sensation triggered by the stimulus. The basic illustration on pain transmission is illustrated in Figure 1.

### 2.1. Neurons

Neurons are known to be a primary component that connects, receives and processes all the nociceptive information generated from the three events discussed above in the CNS and PNS. Three types of neurons that exist in our body are sensory neurons (afferent neurons), interneurons (functions to relay the signals between afferent and efferent neurons) and motor neurons (efferent neurons). All neurons are electrically excitable and consist of the same division of parts: soma, axon (either myelinated or unmyelinated) and dendrites. Neurons are connected with each other to form complex neural networks in our body, where the chemical and electrical signals are transmitted via specialized connections, which are called synapses. The synaptic signals sent from a neuron are received by the dendrites and soma (synaptic transmission) of another neuron, and these signals could be inhibitory or excitatory in nature, defined by the pharmacological effects resulting from the signal itself. After receiving the signals via the dendrites or soma, the signals are transmitted within the neurons by axons. This leads to brief pulses generated within the neuron, known as an action potential, which propagate from the soma, travel along the axons to activate the synapses, and are then sent to other neurons, acting as a pathway to carry the signals from its source to either the spinal cord or the brain, where a response is ultimately interpreted to be executed. There are two major classifications of specialized neurons: sensory neurons and motor neurons. The sensory neurons, which are located in the dermis and epidermis that function to react to stimulus-like touching, send these signals along when the stimulus is present, whereas the main job of the motor neurons is to receive the signals from the brain and spinal cord followed by producing responses causing muscle contractions, and to affect the glandular outputs, as shown in Figure 1. Without the presence of neurons within the nervous system to transmit signals, our body cannot react to dangerous stimuli from the environment.

### 2.2. Axons

Axons are also known as nerve fibers, which are the main component of a neuron that functions to conduct action potentials in a unilateral direction from the dendrites to the axonal terminals, as well as from one neuron to another. Axons can be in the myelinated or unmyelinated form. The presence of the myelin sheath of an axon, known as the node of Ranvier, increases the propagation speed of the impulses, as they travel along the myelinated fiber via saltatory conduction (generation of action potential at each node of Ranvier) and acts as an insulator to prevent electrical impulses from leaving the axon during the transmission. For unmyelinated fibers, the impulses move continuously in a much slower pace, as compared to myelinated ones. For primary afferent neurons, the Aδ-fibers are myelinated, whereas the C-fibers are unmyelinated. For efferent neurons, most of the preganglionic neurons are myelinated. Furthermore, there are small gaps between the nodes of Ranvier. The node of Ranvier contains a K^+^ and Na^+^ channel, which acts as a reserve to save energy of the neuron during action potential transmission. Based on the velocity of a conducted impulse, axon’s diameter, and function of an axon, the sensory (afferent) neurons are classified into three main groups—Group A, B and C, while the motor (efferent) neurons are grouped into Type Ia, Ib, II, III and IV [2].

#### 2.2.1. Group A

Group A nerve fibers were classified by Erlanger and Gasser as fibers that are myelinated. It can be further subdivided into Aα, Aβ, Aγ and Aδ with different sets of characteristics each. These fibers generally terminate in laminae I, III, IV and V of the DH of the spinal cord with some lamina II inner projection.

- Type Aα: both Type Ia and Ib of the sensory fibers from muscle spindle endings and Golgi tendon are grouped into this type. It is mainly used to determine the proprioceptive function.
- Type Aβ: it is a low-threshold, cutaneous, slow or fast adapting type of mechanoreceptors, and is a Type II afferent fiber from the stretch receptor [2]. The Aβ-fibers belong to laminae III and IV.
- Type Aγ: Type II afferent fibers from the stretch receptors.
- Type Aδ: it is well-known as the thermal and mechanical nociceptors that terminate in the rexed laminae I and V [3]. It is a Type III afferent fiber [4]. Aδ-fibers are also the smallest myelinated nerves and have a relatively fast conduction velocity of 30 m/s. The diameter of Aδ-fibers is about 2–5 µm, and is responsive towards short-lasting and pricking pain.

#### 2.2.2. Group B

These groups of nerve fibers are moderately myelinated with conduction velocities of 3–14 m/s. The preganglionic nerve fibers of the autonomous nervous system (ANS) and general visceral afferent fibers belong to this group.

#### 2.2.3. Group C

Group C nerve fibers are unmyelinated with less than 2 µm in diameter and have a relatively slow conduction velocity of approximately 2 µm/s. The nerve fibers at the dorsal roots (Type IV afferent fibers) and postganglionic fibers in the ANS can be categorized in this group. All these fibers are mainly nociceptive in function, carrying the sensory information and assembling around 70% of the afferents nociceptive information, which then enters the spinal cord. C-fibers terminate in laminae I and II in the grey matter of the spinal cord [3]. In terms of nociception, C-fibers nociceptors are polymodal, which are activated by thermal, mechanical and chemical stimuli. The activation of C-fibers is from poorly localized stimuli, such as burning sensation of the skin. In terms of neurochemistry, C-fibers can be classified as either peptidergic or non-peptidergic, and about 50% of these fibers express neuropeptides inclusive of calcitonin gene-related peptide (CGRP), neurokinins and substance P (SP).

Generally, the electrical impulses that travel along the axons can be projected in two ways—the afferent or efferent nerve fibers. In the PNS, afferent nerve fibers are referred to as sensory neurons, of which axons carry the sensory information from regions of the body to the spinal cord, whereas the efferent nerve fibers in the PNS are preganglionic and postganglionic motor neurons that carry the impulses of motor-movement signals out from the spine to the peripheral effectors organs, which include the skeletal muscle and smooth muscles, as shown in Figure 1. In the PNS, the afferent neurons somas are located in the ganglia, and their axons transmit the electrical impulses from ganglion to ganglion and eventually back to the spine. The axons of sensory neurons that are located in the dorsal root are mainly responsible for transducing the somatosensory information via interaction with somatosensory receptors. Somatosensory is a complex sensory neuron pathway that mainly responds to external changes, namely, surface touch, auditory, and visual stimuli. The somatosensory receptors can be activated by these different stimuli that act on mechanoreceptors (including proprioception), nociceptors, thermoreceptors and chemoreceptors.

### 2.3. Action Potential

**Figure 2:**
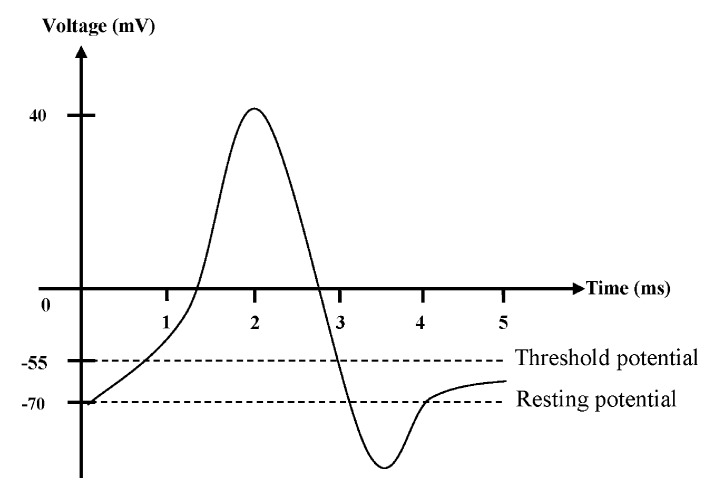
The action potential in neurons.

The release of the neurotransmitters at the axon terminal is triggered upon the entrance of Ca^2+^, and the nociceptive signals are then carried and sent across different neurons by an action potential. There are two major potentials to play unique roles in the production of action potential that allows transmission of signals through the neurons. These are known as the resting potential and threshold potential of neurons. In the axon of a typical neuron, the resting potential and threshold potential are approximately −70 and −55 mV, respectively. There are more Na^+^ accumulated outside the cell than the K^+^ inside the cell, and thus the resting potential of the cells is negatively charged. The movement of these ions across the lipid bilayer membrane of the neurons is strictly dependent on the activation of different ion channels. The conformation of the ion channels can be changed in order to be activated or inactivated, thus allowing for the influx or efflux of specific ions. The action potential in neurons is illustration in Figure 2.

Once the nociceptors are stimulated by a noxious stimulus, two types of potential are generated and summated in the axon hillock, the inhibitory postsynaptic potentials (IPSP) and excitatory postsynaptic potentials (EPSP). Once the triggering threshold is reached, the action potential is then propagated through the axon along the neurons. Generally, the action potential starts, when Na^+^ enters through a voltage-activated Na^+^ channel (Na_v_), which creates the depolarizing nature of the membrane potential. When threshold potential is achieved, all the Na_v_ channels that are located in the axon hillock are stimulated to open, leading to a complete depolarization till achieving peak potential (+40 mV) of the neurons. At this point, the Na_v_ channels return to their resting state, and the voltage-activated K^+^ channels (K_v_) are activated and opened to allow the efflux of K^+^, causing repolarization of the neurons. The shape of the action potential is stereotypical, which means that the amplitude and the time course for all the action potentials occurring in the cells are the same. The continuous efflux of K^+^ through the K_v_ and K^+^ leakage channels causes the membrane potential to hyperpolarize, during the supposedly refractory period of the neurons. Eventually, the K^+^ channels close and the Na^+^/K^+^ transporters restore the resting potential by allowing the entrance of three Na^+^ and exit of two K^+^. When the action potential travels to the axon terminal, the Ca^2+^ enters into the presynaptic terminal through the voltage-operated Ca^2+^ channels (VOCC), hence causing the synaptic transmission [5,6].

### 2.4. Synaptic Transmission

Synaptic transmissions are chemical events used to transmit the impulse between neurons. There is a gap between the presynaptic and postsynaptic membranes, known as the synaptic cleft, where the chemical synapse occurs. The synaptic transmission begins with the arrival of the action potential at the presynaptic axon terminal. An action potential at the presynaptic terminal creates membrane depolarization, which causes the opening of Na_v_ channels at the terminal. The entry of Na^+^ leads to further activation of the VOCC, allowing Ca^2+^ to enter into the axon terminal. These calcium ions bind to the calcium-sensing protein present at a said terminal, which subsequently interact with soluble *N*-ethylmaleimide-sensitive-factor activating protein receptor (SNARE) proteins. The primary role of the SNARE proteins is to promote the fusion of the synaptic vesicles (also known as neurotransmitter vesicles) to the presynaptic axon terminal membrane in the neurons, which causes the immediate release of their contents including neurotransmitters and Ca^2+^ into the synaptic cleft via exocytosis. Free neurotransmitters diffuse across the synaptic cleft and bind to their cognate ligand-gated ion channels that are located on the membrane of the adjacent postsynaptic neuron, causing a localized action potential at the axon of the second neuron. The impulse signals in this pathway can be passed from one neuron to in a unilateral direction, as shown in Figure 1.

There are four possible ways to terminate the release of neurotransmitters. These methods include: (1) drift of the neurotransmitters away from the synaptic cleft after dissociating from its receptors; (2) removal of the neurotransmitters by the astroglia (glial cells) found in the synaptic cleft; (3) enzymatic degradation of the neurotransmitters; and (4) re-uptake of the neurotransmitters into the axon terminal that releases them.

### 2.5. Route of Pain Transmission

Fundamentally, pain transmission is strictly dependent on the balance of the excitatory and inhibitory influences that act on the neuron circuits of the somatosensory system. There are multiple levels of CNS involved in the transmission of pain. These include the spinal cord (supraspinal), the brainstem (midbrain, medulla oblongata and the pons), and the cortical regions (cerebral cortex), as shown in Figure 1. Typically, the DH of the spinal cord plays a crucial role in integrating multiple inputs entering the spine, including the primary afferent neurons and local interneuron networks, and is also responsible for the descending signals from the supraspinal center.

Within the ascending system, primary afferent nociceptors are responsible for conveying the noxious information received to the projection neurons in the DH of the spinal cord. Following that, a subset of these projection neurons in turn transmit these sensory information up to the thalamus reaching the somatosensory cortex through the spinothalamic tract, thus providing information on the intensity and the location of the noxious stimulus. The spinothalamic tract is located in the white matter of spinal cord and consists of two parts—the lateral spinothalamic and anterior spinothalamic tracts, which have different courses of function. The lateral spinothalamic tract focuses on transmission of the pain and temperature sensation, while the anterior spinothalamic tract carries information related to the crude touch and firm pressure sensation towards the thalamus in the brain.

Other projection neurons engage the cingulate and insular cortices via the connections in the parabrachial nucleus and the amygdala, hence contributing to the pain experiences. As shown in Figure 1, this ascending information accesses the neurons of the periaqueductal gray (PAG) and rostral ventral medulla (RVM) that is found in the midbrain to engage the descending feedback systems, in order to regulate the output from the spinal cord [4]. The core function of the PAG is to integrate the information received from the higher centers of the brain, including the hypothalamus, amygdala and frontal lobe, as well as receiving the ascending nociceptive input from the DH. The PAG regulates the processing of the nociceptive information in the DH of the spinal cord via the projection neurons to RVM and dorsolateral pontine tegmentum (DLPT). The endogenous opioid and cannabinoid systems and other neurotransmitters, such as 5-hydroxytryptamine (5-HT) and norepinephrine (NE), are heavily expressed through the PAG/RVM pathways.

## 3. Types of Pain

Typically, pain can be classified into three types—nociceptive, neuropathic and inflammatory pain, based on three characteristics, such as symptoms, mechanisms and syndromes.

### 3.1. Nociceptive Pain

Nociception used interchangeably with nociperception is the response of our bodies’ sensory nervous systems towards actual or potentially harmful stimuli. The sensory endings that are activated by such stimuli are known as nociceptors, which are mainly responsible for the first stage of pain sensations. Fundamentally, the Aδ- and C-fibers are two types of primary afferent nociceptors responding to noxious stimuli presented in our bodies’ [7]. Both these nociceptors have specialized free nerve endings that are widely located in the skin, muscle, joint capsule, bone and some major internal organs. They are functionally used to detect potentially damaging chemical, mechanical and thermal stimuli that might put us in harm’s way.

The major nociceptive pain can be categorized into two types including visceral somatic pain (which is further classified into two kinds: deep somatic and superficial pain). Both the Aδ- and C-fibers are mostly found in superficial organs, such as the skin, whereas other deep somatic structures, such as muscles and joints, are mainly supplied with C-fibers. Aδ-fibers are activated under thermal or mechanical stimuli and result in a short-lasting-pricking type of pain sensation. However, the activation of C-fibers is stimulated by thermal, mechanical or chemical stimuli, which often results in poor localization and dull pain sensation. There are three major roles for the receptors in the primary afferent neurons, which are excitatory, sensitizing and inhibitory response. Once these receptors are being stimulated and have reached the pain threshold, the resulting impulses are propagated along the afferent fibers towards the DH (PNS) and medulla (cranial). On top of that, there is an additional nociceptor known as silent nociceptors. Silent nociceptors are located within the viscera and these afferent nerve fibers have no terminal morphological specializations without responses to noxious stimuli, but can only be sensitized by the chemical mediators produced during inflammatory reactions.

### 3.2. Neuropathic Pain

Neuropathic pain is commonly described as a nerve injury or nerve impairment and is often associated with allodynia. Alloydnia is a central pain sensitization that is a result of repetitive non-painful stimulation of the receptors. It triggers a pain response from a stimulus that is deemed as non-painful in normal conditions, due to sensitization process from said repetitive stimulation. This condition can be described as “pathologic” pain, because neuropathic pain actually serves no purpose in terms of defense system for our body, and the pain could be in the form of continuous sensation or episodic incidents. The major causes of this type of pain could be primarily due to inflammation or metabolic diseases, such as diabetes, trauma, toxins, tumors, primary neurological diseases and herpes zoster infection. The central sensitization plays a rather important role in this process. Neuropathic pain can be caused by the damage of the nerve, affecting the somatosensory nervous system, and may be generated by the disorders of the PNS or CNS.

The neurochemistry of the damaged axons can be altered due to the initiations of complex reaction upon compression, stretching, or transaction of the periphery nerves, followed by a spontaneous hyper-excitability on the site. During neuropathic pain, nociceptors demonstrate a dynamic expression of ion channels, such as Na_v_ channels. In fact, Na_v_ channels are the major channels in regulation of the neuronal excitability, initiation and propagation of the action potentials. The Na^+^ current in the dorsal root ganglion (DRG) can be classified into three types, namely, fast tetrodotoxin-sensitive (TTX-S), slow tetrodotoxin-resistant (TTX-R) with high-activation thresholds and persistent TTX-R with lower activation thresholds [8]. TTX is a potent neurotoxin and acts as a Na_v_ channel blocker whereby its binding with the Na_v_ channels inhibits the firing of action potentials generated in the neurons [9].

### 3.3. Inflammatory Pain

Inflammation is a natural biological response produced by the tissues within our body as a reaction to the harmful stimuli in order to eradicate the necrotic cells and initiate the tissue repairing process. Neutrophils are usually the first respondents of an inflammatory response and gather at the site of injury via the bloodstream, followed by the release of other chemical mediators [10]. Inflammation may lead to three major responses: hyperalgesia, allodynia and sympathetic maintained pain. An inflammation can also induce mast cell degranulation, which subsequently leads to the release of platelet activating factor (PAF) and stimulates the release of 5-HT from the circulating platelet. The cardinal signs of inflammation include the hot inflamed site due to increase in blood flow towards the region, redness, and swelling due to vascular permeability pain caused by the activation and sensitization of primary afferent neurons and lasting loss of function. The localized inflammatory response then induce the release of free arachidonic acid (AA) from the phospholipids, which are converted into prostaglandins (PG) via the cyclooxygenase (COX) pathways.

Pain from inflammation can be further classified into two types: chronic and acute pain. Acute inflammatory pain is normally intense and occurs for a short period of time, which is initiated as a response to harmful stimuli that are normally mediated by the Aδ-fibers. Leukocytes and plasma from the bloodstream are accumulated at the site of the injury to assist in the inflammatory process. However, prolonged inflammation, better known as chronic inflammatory pain, lasts beyond the expected period of healing, which is typically mediated by C-fibers [4]. There is a progressive shift of mononuclear cells at the site of the inflammation as well. Inflammatory pain causes the increase of afferent input into the DH of the spinal cord and leads to the development of central sensitization. There are some mediators produced at the site of injured tissue, which include 5-HT, kinins, histamine, nerve growth factors (NGF), adenosine triphosphate (ATP), PG, glutamate, leukotrienes, nitric oxide (NO), NE and protons [11]. During the process of inflammation, these chemical inflammatory mediators are produced from the necrotic tissues, and interact to activate the nociceptors within the inflamed area.

### 3.4. Arthritis

Arthritis in layman terms can be defined as joint inflammation. The major causes of arthritis include bone erosion, formation of new bones, synovial hyperplasia, ankylosis of the joint and infiltration of inflammatory cells. The cardinal signs involved include redness, swelling, hotness, and large reduction in the range of motion of the affected joints. There are currently more than a hundred types of arthritis that patients suffer from. Among them, osteoarthritis, rheumatoid arthritis and gout are easily described as the most common type of arthritis reported. Osteoarthritis often occurs in patients with advanced age due to the degeneration of joint cartilage or its underlying bone. Its pain is well-localized and occurs during weight-bearing movement, whereas rheumatoid arthritis is an autoimmune disease of the synovium that leads to polyarthritic conditions. It commonly affects our hands or feet. Gout is one of the most painful forms of arthritis, which is caused by the persistent elevation of uric acid in the bloodstream, leading to significant presence of crystal formation in the joints, tendons and surrounding tissues. It commonly occurs in those who are regularly consuming red meat and beer. Along with the inflammation of joints, pain is an accompanying factor in patients suffering from arthritis, especially during movements due to its restrictions.

## 4. Hyperalgesia

Hyperalgesia is a natural phenomenon that refers to tenderness or lowered threshold to the thermal or mechanical stimulation-induced pain (primary hyperalgesia). This results in an enhanced perception of pain at the site of injury [12]. The pain messengers, such as cytokines and chemokines, are distributed to chemical receptors at and around the trauma site to cover a larger area than the actual injured region. PG is the major component for sensitizing procedure of the nociceptors. Due to pain messengers attaching to receptors around the injury site, it causes the sensitization of the adjacent uninjured tissue to the mechanical stimuli, which is commonly known as secondary hyperalgesia or allodynia. The primary hyperalgesia has a major peripheral component, while secondary hyperalgesia is due to central sensitization and mediating mechanisms residing within CNS.

## 5. Allodynia

Allodynia refers to central sensitization that leads to the triggering of pain response that normally does not provoke pain, such as a light touch [7,13]. The cells involved in the mechanical sensation and nociception are those responsible for allodynia. Upon peripheral nerve injury, the anatomical reorganization occurs whereby sprouting the A-fibers into lamina II in the DH of the spinal cord, which originally receives the nociceptor input from C-fibers. Both hyperalgesia and allodynia occur due to the increase of prostaglandin E_2_ (PGE_2_) in the inflamed tissue via the activation of COX signaling pathway in the DH of the spinal cord [14].

## 6. Peripheral Sensitization

The repetitive exposure to noxious stimuli triggers an action potential to be propagated to the central terminal via the sensory neurons, as well as to the peripheral terminal via the collateral axon branches, and this subsequently causes the membrane depolarization along with Ca^2+^ influx via the VOCC, which in turn induces the transmitters to be released at the site of the injury and activates the surrounding nociceptors. This process is known as sensitization. Sensitization is described as the decrease in threshold to stimulation, as well as an increase of firing rate due to the enhanced sensitivity of primary afferent nociceptors. In fact, this enhanced and prolonged response to the stimuli can be manifested as primary hyperalgesia. The summation of the released intracellular contents, including ATP, bradykinin (BK), 5-HT, NE, PGE_2_, NGF and SP at the site of the damaged cells or inflammatory cells, is also known as inflammatory soup. There is an increase in the concentration of proton (H^+^) at the injury site, thus increasing its acidity. All these inflammatory mediators bind and are activated by their respective cognate receptors that are located on the postsynaptic neurons. and subsequently augment the pain sensation through the interaction among a variety secondary messenger. For instance, the cellular cyclic adenosine monophosphate (cAMP)/protein kinase A (PKA) and protein kinase C (PKC)/diacylglycerol (DAG) signaling activities have been shown to be crucial for maintaining peripheral hyperalgesia, whereas the cyclic guanosine monophosphate (cGMP) plays an opposite role in the cAMP during nociceptor sensitivity [5].

There are other nociception-specific receptors that are present at the afferent terminals: the capsaicin receptor, transient receptor potential cation channel, subfamily V (TRPV1) or vanilloid receptor for capsaicin (VR1) that acts as a transducer for dangerous thermal stimuli. The signaling mechanism pathways involved in the afferent terminal sensitization have included elevation of the Ca^2+^ and activation of G-protein coupled receptors (GPCRs) that results in the elevation of adenylyl cyclase (AC)/cAMP/PKA, phospholipase C (PLC)/inositol triphosphate (IP_3_)/Ca^2+^ or PLC/DAG/PKC activities. On the other hand, the inflammatory mediators can also stimulate the antidromic release of the transmitters from the collateral branches of the afferent nerves, which is commonly known as neurogenic inflammation. Neurogenic inflammation occurs when inflammatory mediators, such as SP, CGRP and neurokinin (NK), are released locally by the afferent neurons. The end result of peripheral sensitization is to generate more primary afferent nociceptors activities, and when this information reaches the DH of the spinal cord, the pain sensation is enhanced even with the strength of the stimulus remains unchanged.

## 7. Central Sensitization

Central sensitization is the repetitive stimulation of the nociceptors that causes amplification in the nociceptive information, leading to the excitability of the projection neurons within the DH of the spinal cord. The DH of the spinal cord usually responds to low intensity of stimuli. When the action potential reaches the presynaptic terminal via the activation of the N-type or P-type VOCC, there are neurotransmitters released from the afferent terminal to produce excitatory effects, including SP and glutamate. In contrast, the inhibitory effects are modulated by γ-aminobutyric acid (GABA), NE, glycine, adenosine, endogenous cannabinoids and opioid peptides.

## 8. Neurogenic-Induced Inflammation

The neurogenic inflammation is mediated by neuropeptides released from the sensory nerve endings. This creates a “flare” reaction when there is a scratch injury. Generally, there are a few neuropeptides that mediate this event, which include SP, NK and CGRP. These neuropeptides activities cause vasodilation and plasma extravasation, hence facilitating the body’s immunity cell’s entry to the site of inflammation and resulting in the development of oedema.

## 9. Major Types of Pain-Mediated Neurotransmitters

**Figure 3:**
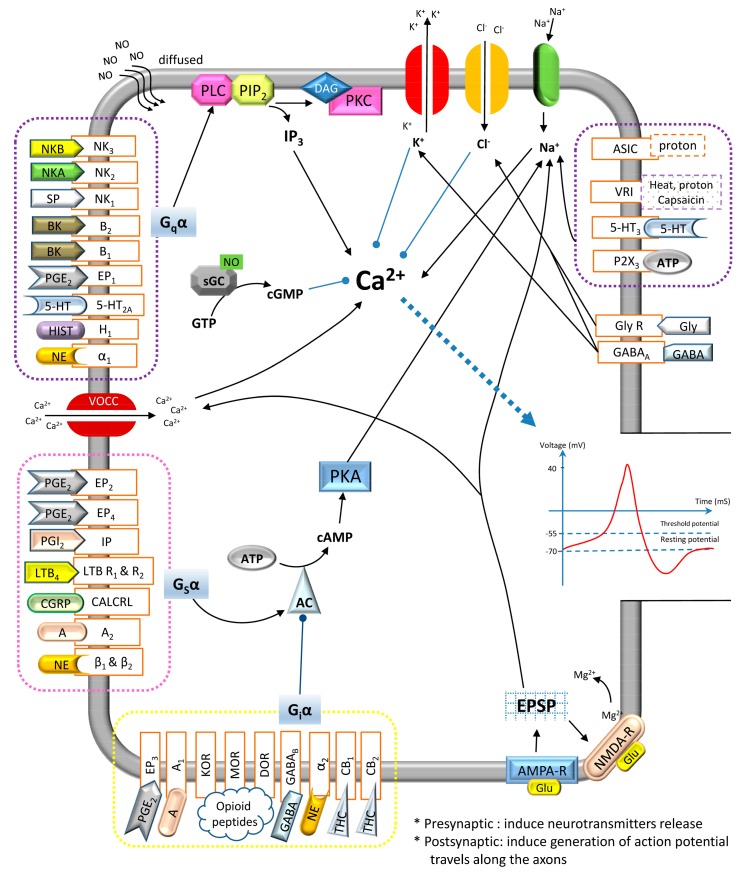
The signaling mechanism pathways of pain-associated neurotransmitters and their cognate receptors involved in pre- and post-synaptic locations for pain transmission. 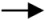: Activate/Enhance production; 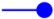: Inhibit/Reduce production; 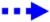: Generate/Lead to.

There are varieties of neurotransmitters involved in the pain sensation. All the major types of neurotransmitters (inflammatory mediators: PGE_2_, PGI_2_, LTB_4_, NGF, proton, BK, ATP, adenosine, SP, NKA, NKB, 5-HT, histamine, glutamate, NE and NO; non-inflammatory mediators: CGRP, GABA, opioid peptides, glycine and cannabinoids), the second messenger production, their interaction with different channel-linked receptors, and resulting pharmacological effects in the pre- and postsynaptic neurons terminal are illustrated in Figure 3. Furthermore, the neurotransmitters’ releasing location in the body, the location of their cognate receptors (either pre- or post-synaptical), and eventual pharmacological effects on pain regulation are further elaborated in Table 1 and Table 2.

### 9.1. Tachykinins

Tachykinins is the largest family of neuropeptides. There are three members of tachykinins family involved in the neurogenic-induced inflammation, which are SP, neurokinin A (NKA) and neurokinin B (NKB). These neuropeptides are produced from peripheral terminals of the sensory nerve fibers, such as muscle and skin via proteolytic cleavage of their precursor, pre-protachykinins [34]. The SP, NKA and NKB selectively bind to their cognate receptor according to their affinity to these receptors. For a clearer picture of the receptors that are compatible with the neuropeptides, SP binds to neurokinin type 1 receptor (NK_1_) while NKA binds to neurokinin type 2 receptor (NK_2_) and NKB binds to neurokinin type 3 receptor (NK_3_) receptors, respectively. All these receptors are G_q_-protein coupled receptors, which mediate through PLC/IP_3_ and DAG/PKC signaling pathways upon activation, hence resulting in its excitatory effects.

### 9.2. Calcitonin Gene-Related Peptide

CGRP is widely produced in both central and peripheral nervous systems; however, it is primarily located in the primary afferent nerves. As a direct derivative of the DRG, CGRP is found in the DH of the spinal cord and associated with the conduction of noxious stimulation [57]. CGRP is related to the excitatory effects of SP, which results in Ca^2+^ release. The receptors of CGRP are G_s_-protein-coupled, which are known as calcitonin receptor-like receptor (CALCRL) located in the nucleus accumbens, indicating that the CNS controls the CGRP-mediated pain transmission [72].

### 9.3. Bradykinin

BK is a well-known algogen and acts as one of the inflammatory mediators that are locally produced from the breakdown of high-molecular-weight kininogens in the site of the inflamed tissue. In the nociceptive afferent nerve fibers, BK binds to G_q_-protein-coupled bradykinin receptor type B_1_ (B_1_) or bradykinin receptor type B_2_ (B_2_) receptors, leading to sensitization. The activation of B_1_ or B_2_ receptors causes activation of the PLC to break down the phosphatidylinositol 4,5-bisphosphate (PIP_2_) into IP_3_ and DAG, and subsequently, DAG activates the PKC, leading to the increase of Ca^2+^ conductance [73,74]. Furthermore, BK can act synergistically with other algogenic substances, such as PG and NGF, to further stimulate the production of pro-inflammatory cytokines. It can also further sensitize the nociceptors, such as interleukin-2 (IL-2), 5-HT, histamine and prostanoids, to heat.

### 9.4. Cytokines

During the degranulation of the inflammation-induced mast cell, the PAF is stimulated for release and subsequently induces the production of serotonin or 5-HT from the circulating platelets. It is an indolamine mediator, which causes extravasation of plasma and hyperalgesia in human and rats. The 5-HT receptors are located on the nerve cells membrane and all of these receptors are GPCRs except the 5-hydroxytryptamine type 3 receptor (5-HT_3_) receptor, which is ligand-gated ion channel [75]. Two major types of 5-HT receptors present on the sensory neuron’s terminal are 5-hydroxytryptamine type 2A receptor (5-HT_2A_) and 5-HT_3_. 5-HT_2A_ receptors are G_q_-protein-coupled, which enhances the pain sensation through PLC/IP_3_ and DAG/PKC pathways, whereas the activation of the 5-HT_3_ receptors induces a depolarization current, hence causing excitatory effects to be produced. In addition to 5-HT_3_, histamine is one of the well-known products of mast cell degranulation, which reacts with its G_q_-protein coupled receptor (H_1_) located at the afferent nerve terminal, aiding in the inflammation process. Additionally, other cytokines, such as interleukin 1β (IL-1β) and tumor necrosis factor α (TNFα), play a crucial role in exerting a powerful pro-inflammatory effect that causes hyperalgesia, as well as in exerting a synergistic interaction upon contact with NGF.

### 9.5. Prostaglandins

PG is produced from the AA via the catalysis of COX. They can be found in other tissue in our bodies and are considered as an archetypal sensitizing agent that reduces the nociceptive threshold as well as the core cause of tenderness. PGE_2_ (produced by cyclooxygenase-2) and prostacyclin (PGI_2_) (produced by cyclooxygenase-1) are two major prostaglandins that lead to a direct afferent sensitization. The receptor of PGE_2_ can be divided into 4 major types, such as prostaglandin E_2_ receptor type 1–4 (EP_1–4_), whereas the receptor of PGI_2_ is termed prostacyclin receptor (IP). EP_1_ is G_q_-protein coupled receptor, which leads to the PLC/IP_3_ and DAG/PKC signaling pathway, EP_2_, _4_ and IP are G_s_-protein-coupled receptors, which act on promotion of the AC/cAMP/PKA signaling pathways upon activation, whereas EP_3_ is a G_i_-protein-coupled receptor, which leads to inhibitory effects. In addition, PG enhances the effects of other chemical mediators, such as 5-HT and BK, as well as augments the neuropeptide, such as SP and CGRP, to be released. In other words, the increase of the BK induces the PG to be released and results in a “self-sensitizing” effect.

### 9.6. Leukotriene B_4_

LTB_4_ is one of the eicosanoid inflammatory mediators that are produced within the leukocytes. Upon injury caused by mechanical or thermal stimuli, the AA is broken down into 5-hydroperoxyicosatetraenoic acid (5-HPETE) by lipooxygenase (LOX), and is subsequently hydrolyzed into LTB_4_ by leukotriene A_4_ (LTA_4_) hydrolase [76]. LTB_4_ is mainly responsible for recruiting neutrophils towards the site of the damaged tissue, whilst simultaneously promoting the production of cytokines [77]. In the presence of polymorphonuclear (PMNs) leucocytes, LTB_4_ can indirectly cause hyperalgesia probably through the afferent terminal pathway [19]. LTB_4_ can cause sensitization of the nociceptors by increasing the cAMP/PKA activities. Some animal studies have speculated that the accumulation of inflammation-induced neutrophil is highly associated with the increasing number of LTB_4_, which causes the indirect stimulation of hyperalgesia.

### 9.7. Proton

The site of injury is often more acidic than homeostasis, and hence the local content of protons shows a hike in number. The increasing number of these protons activates both the acid-sensing ion channels (ASICs) and VR1 around the injury location. ASICs are neuronal voltage-insensitive Na^+^ channels, which are activated by extracellular protons. Typically, these channels respond to a low pH surrounding and are suggested by studies to be involved in the modulation of our bodies’ mechano-sensation [78]. Furthermore, there was research indicating the exposure of primary afferent nerve fibers to a pH lower than 6 can stimulate the ASICs [79]. VR1 can also be activated by these protons (H^+^, pH < 5.5) via either heat stimuli or capsaicin. VR1′s (a member of TRPV group of transient receptor potential family of ion channels) location in the dorsal roots of primary afferent nerves makes it mainly responsible for detection and regulation of the body’s temperature, thus providing a burning sensation when stimulated by heat [80]. Upon the activation of both the VR1 and ASICs, the presence of BK, PGE_2_ and histamine at the injury site can further increase the intracellular Ca^2+^ influx, hence enhancing the expression of VR1 and sensory neuron-specific (SNS) Na^+^ channels. Subsequently, the influx of the Na^+^ generates an action potential, thus causing sensitization of the afferent nerves. Although the rise of the intracellular Ca^2+^ leads to the release of the SP and CGRP, it can desensitize VR1 [81,82].

### 9.8. Adenosine Triphosphate

ATP is an important intracellular messenger that is released locally by the damaged tissues and directly stimulates its receptors. This occurs when ATP is metabolized into adenosine by ectonucleotidases and binds to its receptor, ionotropic purino receptors (P2X) that are located at the peripheral site of the sensory neurons and centrally on the second-order neurons in the DH [30,32]. In general, there are six types of P2X receptors, including P2X_1–6_ expressed in the sensory neurons. Amongst these six types, purino receptor type 3 (P2X_3_) receptors are one of the most selectively expressed receptors in the small C-fibered nociceptor. Once the ATP binds to the P2X_3_ receptors, Na^+^ can cross these channels and induce membrane depolarization, hence activating various Ca^2+^-sensitive intracellular processes and causing both pain and hyperalgesia. ATP can presynaptically act on the nociceptors to increase the release of glutamate. On the other hand, ATP produces a by-product from its metabolism, adenosine, which binds to either adenosine type 1 receptor (A_1_) G_i_-PCRs for inhibitory action or binds to the adenosine type 2 receptor (A_2_) G_s_-PCRs that are located peripherally and centrally to sensitize the nociceptors via the cAMP/PKA signaling pathways.

### 9.9. Nerve Growth Factor

The NGF is a neurotrophic factor of neuropeptide—a well-known mediator for persistent pain, which is locally released at the site of injury by fibroblasts. After the inflammatory lesions are formed, the NGF is expressed rapidly and can induce a rapid onset of mechanical and thermal hyperalgesia. NGF-dependent nerve fibers, known as tropomyosin receptor kinase A (TrkA), are also a high-affinity NGF receptor. This receptor is widely expressed in the primary afferent neurons with up to 50% coverage. which suggests its direct role in activation for peripheral sensitization. In addition, there are some non-neuronal cells including keratinocytes, mast cells and circulating eosinophils capable of expressing TrkA receptors and responding to the injury-induced NGF. For instance, NGF can cause mast cells degranulation and the release of 5-HT and histamine, as well as positively stimulate the release of more NGFs to enhance the inflammatory signals.

### 9.10. Glutamate

Glutamate is the most abundant excitatory neurotransmitters in the vertebrate nervous system [83] that presents itself at the periphery inflammation sites, as well as contributes to over 50% of the brain synapses. It plays a crucial role in modulating nociception and neurotransmitter released from the terminal afferents. Theoretically, the sensory neurons express a full complement of the glutamate receptors, including alpha amino-3-hydroxy-5-methyl-4-isoxazolepropionic acid receptors (AMPA-R) and *N*-methyl-d-aspartate receptors (NMDA-R). Whilst it can be released by both the low- and high-threshold sensory afferent fibers to activate the C-fibers, these actions highlight the feedback mechanism, where the nociceptor excitability is enhanced by its own activity and probably causes an auto-regulation. The receptor of glutamate is NMDA-R, an ion channel that requires a specific combination of events in order to be activated, and results in the increase of the intracellular Ca^2+^, causing central sensitization [83].

Upon being stimulated, glutamate is released by the sensory afferent fibers and acts via the AMPA-R, hence generating a rapid EPSP. The AMPA-R is a sodium-selective ion channel and its activation by the glutamate or amino-3-hydroxy-5-methyl-4-isoxazolepropionic acid (AMPA) can subsequently generate a short-lived post-synaptic depolarization. After repeated stimulation, other tachykinin neuropeptides, such as SP, NKA and NKB, are co-released with glutamate and react with their receptors, such as NK_1_, NK_2_ and NK_3_, respectively, in which EPSP is prolonged. SP, NKA and NKB are grouped as tachykinin peptides with CGRP, predominating in the DH as excitatory peptide transmitters. This EPSP produces a sustained membrane depolarization, causing the Mg^2+^ that has blocked the pore of NMDA-R channel to be removed. In fact, NMDA-R is blocked by Mg^2+^ at normal physiological membrane potential. At this stage, the glutamate and glycine bind to activate the NMDA-R, resulting in calcium ion influx and causing hyperexcitability in the postsynaptic neurons [84].

Furthermore, there are various effectors that can indirectly enhance the activation of NMDA-R. For instance, the intracellular Ca^2+^ influx through the NMDA-R causes a feedback to this receptor in order to increase the number of channels that are open. The increasing activity of the cAMP/PKA pathways via the production of PGE_2_ can further enhance the function of NMDA-R. NO can be produced by neuronal NO synthase (nNOS) from the l-arginine in the spinal cord neurons in CNS [54]. Unlike other neurotransmitters, NO is mobile, free to diffuse from the neurons and enter other neurons to produce its second messenger through an enzymatic reaction. NO plays a crucial role in the transmission of nociceptive information after an inflammatory reaction, whilst stimulating the activation of NMDA-R. In short, the key second messengers mediated through activation of NMDA receptors are inclusive of cAMP, PKA, Ca^2+^ and PKC, which subsequently cause the increase of excitability of the DH neurons to respond towards weaker afferent nociceptive inputs.

### 9.11. γ-Aminobutyric Acid (GABA)

GABA is the most widely distributed inhibitory transmitter in a mammalian CNS. It contributes to about 40% of our brain synapses and can be found in the interneurons of the spinal cord, neocortex and cerebellum [85]. It is produced by GABAergic neurons, which are concentrated in the brain. Within the nervous system, GABA can bind to the ionotropic GABA_A_-receptors or metabotropic GABA_B_-receptors, since they are widely found in the nervous system with GABA_B_ and concentrated at the presynaptic nerve terminals as well as in the CNS. However, GABA_A_ receptors are generally the receptor of choice for binding of GABA in the CNS because it is largely located there. When GABA binds to GABA_A_ receptors, there is an inflow of extracellular Cl^−^ into the neurons, thus reducing the membrane potential and resulting in an inhibitory effect. On the other hand, the binding of GABA to GABA_B_ receptors causes an inhibition towards the formation of cAMP, because GABA_B_ receptor is a G_i_-protein-coupled receptor [60].

### 9.12. Opioid Peptides

The peptides that bind to the opioid receptors, such as µ-opioid receptors (MOR), δ-opioid receptors (DOR) and κ-opioid receptors (KOR), are known as opioid peptides. All classes of the opioid receptors are G_i_-protein-coupled receptors, which means they inhibit the AC/cAMP activity upon their activation. These receptors are widely distributed in both the primary afferent neurons and the dendrites of postsynaptic neurons, and there are two endogenous opioid peptides abundantly released into the interneurons of the DH: enkephalin and dynorphin. These peptides inhibit the release of excitatory neurotransmitters from the afferent terminals, hence reducing the excitability of neurons and overall mitigation of the pain sensation as an end result.

### 9.13. Cannabinoids

Cannabinoid is one of the classes in the neurotransmitters that binds itself to its receptors and modulates the neurotransmitters released in the brain. The most common type of cannabinoid is the tetrahydrocannabinol (THC), which is one of the major psychoactive components isolated from *Cannabis sativa*. Cannabinoids can bind to G_i_-protein coupled cannabinoid type 1 receptors (CB_1_), which is highly expressed in the pre- and post-synaptic in brain and spinal cord [86,87], as well as the G_i_-protein-coupled cannabinoid type 2 receptors (CB_2_) that is predominantly located in the immune system [69]. The activation of CB_1_ and CB_2_ inhibits the formation of intracellular cAMP, hence leading to a tremendous reduction of the excitatory effect within the neurons [88,89]. In addition, the activation of CB_2_ can further prevent the mast cell degranulation and the release of pro-inflammatory mediators, making the reduction in pain sensation even more drastic and effective.

### 9.14. Norepinephrine

NE is the principal neurotransmitter of the adrenergic pathways and is synthesized from phenylalanine in the nerve terminals. Phenylalanine is converted into tyrosine and then into 3,4-dihydroxyphenylalanine (DOPA) by tyrosine hydroxylase. DOPA is then further converted into dopamine, which is the precursor of NE that is stored in the vesicles of the nerve terminals. The receptors of NE include α1-G_q_α-, α2-G_i_α-, β-G_s_α-protein-coupled receptors. α1-G_q_α- and β-G_s_α-protein-coupled receptors are predominantly located in postsynaptic neurons, whereas α2-G_i_α-protein-coupled receptors are located in presynaptic neurons. Thus, the activation of the α2-G_i_α-protein-coupled receptors inhibits the Ca^2+^ influx, and causes the reduction of NE release out from the synapse. On the other hand, the binding of NE with α1-G_q_α- and β-G_s_α-protein-coupled receptors that are located in the postsynaptic neurons stimulates the PLC/PKC and cAMP/PKA signaling pathways, respectively, and causes excitatory effects.

## 10. Conclusions

In conclusion, understanding the complex mechanisms of pain is undoubtedly essential for pain research and pain management. Hence, the present review was comprehensively discussed based on the molecular and cellular mechanisms underlying the pain pathway as a whole picture. Moreover, the major types of neurotransmitters involved in the pain transduction, transmission and modulation have been completely elaborated along with their locations and eventual pharmacological effects. This could enlighten the understanding of the global scientists towards the pain topic and provide a useful guide for continue analgesic drug discovery in future.

## Figures and Tables

**Table 1:** The pain-associated neurotransmitters releasing location, their cognate receptors’ locations, signaling mechanism pathways involved, agonists and pharmacological effects involved in the pain regulation.

Neurotransmitters	Locations of Chemicals	Receptors: Mechanisms	Receptors’ Locations (Pre-/Post-Synaptically)	Agonists	Pharmacological Effects	Mediate Indirectly	References
*Inflammatory Mediators*							
PGE_2_ & PGI_2_ (Eicosanoid)	CNS & PNS	EP_1_: PLC/IP_3_, DAG/PKC IP, EP_2_, EP_3_ & EP_4_: AC/cAMP/PKA	EP_1–4_: CNS, PNS (DRG of sensory neurons, mast cells, pulmonary veins, colon smooth muscle) IP: CNS (brain), PNS (thymus, VSMC, VEC, DRG in sensory neuron) (+/+)	EP_1–4_: PGE_2_, PGE_1_ IP: Prostacyclin	Excitatory (IP, EP_1_, EP_2_ & EP_4_)/inhibitory (EP_3_)	1. Sensitize VR1 and SNS Na_v_ receptors 2. Augment the release of SP, IL-2, histamine, 5-HT, bradykinin and CGRP	[15,16,17]
LTB_4_ (Eicosanoid)	PNS	LTB_4_-R_1_ & LTB_4_-R_2_: AC/cAMP/PKA or PLC	LTB_4_-R_1_ & LTB_4_-R_2_: PNS (nociceptive afferent neurons) (−/+)	LTB_4_	Excitatory/ inhibitory	1. Sensitize nociceptors 2. Recruit neutrophils to injury site 3. Promote the cytokines production	[18,19]
NGF (Neuropeptide)	CNS & PNS	TrkA: PI3/Ras	TrkA: PNS (primary afferent neurons) (−/+)	NGF, Neurotrophin	Excitatory	1. Cause the mast cells degranulation2. Augment the release of 5-HT, histamine and itself	[20,21]
Proton	CNS & PNS	ASIC & VR1: Na^+^/K^+^	ASIC: CNS (DH of spinal cord), PNS (sensory neurons) (−/+) VR1: CNS, PNS (dorsal root of primary sensory neurons) (+/+)	ASIC: Protons VR1: Heat, capsaicin and protons	Excitatory	1. Enhance the release of BK, SP, CGRP, histamine and PGE_2_	[22,23,24,25]
BK (Neuropeptide)	CNS (pituitary and hypothalamus) & PNS	B_1_ & B_2_: PLC/IP_3_, DAG/PKC	B_1_: CNS, PNS B_2_: CNS (cerebral cortex, hippocampus and spinal cord), PNS (nociceptive afferent neurons) (+/+)	BK	Excitatory (B_1_ & B_2_)	1. Augment the release of PG, NGF and pro-inflammatory cytokines (IL-2). 2. Exert synergistic interaction with NGF and PG	[26,27,28,29]
ATP & Adenosine (Purine)	CNS & PNS	P2X_3_: Na^+^/ K^+^ A_1_ & A_2_: AC/cAMP/PKA	P2X_3_: CNS, PNS (nociceptive afferent neurons especially C-fibers) A_1_: CNS (basal forebrain), PNS (VSMC) A_2_: CNS (basal ganglia), PNS (vasculature, platelets) (+/+)	P2X_3_: ATP A_1_ & A_2_: Adenosine	Excitatory (P2X_3_ & A_2_)/inhibitory (A_1_)	1. Sensitize the nociceptors 2. Enhance glutamate release	[30,31,32,33]
Tachykinins: SP, NKA and NKB (Neuropeptides)	CNS (predominant in DH of spinal cord) & PNS (from C-fibers)	NK_1_, NK_2_ & NK_3_: PLC/IP_3_, DAG/PKC	NK_1_: CNS (brainstem, spinal cord), PNS (VEC, muscle, immune cells)NK_2_: CNS (cingulated cortex, amygdale, prefrontal cortex)NK_3_: CNS, PNS (uterus, mesenteric vein, placenta) (−/+)	NK_1_: SP NK_2_: NKA NK_3_: NKB	Excitatory	1. Activation of NOS and AA pathways for the release of NO and PGE_2_, respectively 2. Enhance the cAMP/PKA activities 3. Mediates neurogenic inflammation	[34,35,36,37,38,39]
5-HT	CNS & PNS (platelet/GI)	5-HT_2A_: PLC/IP_3_, DAG/PKC 5-HT_3_: Na^+^/ K^+^	5-HT_2A_: CNS (neocortex, olfactory tubercle), PNS (sensory neurons) 5-HT_3_: CNS (hippocampus, neocortical interneurons, amygdale), PNS (sensory neurons) (+/+)	5-HT	Excitatory	1. Exert synergistic interaction with NGF	[40,41,42,43,44]
Histamine (Monoamine)	CNS & PNS	H_1_: PLC/IP_3_, DAG/PKC	CNS, PNS (VSMC, VEC, sensory nerve) (+/−)	Histamine	Excitatory	1. Exert synergistic interaction with NGF	[45,46]
Glutamate (Amino acid)	CNS (abundant) & PNS (in C-fibers)	AMPA-R & NMDA-R: Mg^2+^/Ca^2+^/Na^+^/K^+^ (EPSP) *NMDA-R need both glutamate/aspartate & co-exist of glycine to be activated	AMPA-R: CNS NMDA-R: CNS, PNS (nociceptive sensory neurons) (−/+)	AMPA-R: AMPA, glutamate NMDA-R: Glutamate, alanine, aspartate with co-exist of glycine [47]	Excitatory		[48,49,50,51]
NE (Monoamine)	CNS & PNS	α1: PLC/IP_3_, DAG/PKC α2: AC/cAMP/PKA β: AC/cAMP/PKA	α1: CNS (brain), PNS (VSMC, GI, kidney) (−/+) α2: CNS (predominant), PNS (+/−) β: CNS (cerebral cortex), PNS (cardiac tissues) (+/+)	NE/Epinephrine/Isoprenline	Excitatory (α1 & β)/Inhibitory (α2)		[52,53]
NO (Gasotransmitter)	CNS & PNS	sGC/cGMP	-	-	Excitatory/Inhibitory	1. Recruited to the site of inflamed tissue	[54]
*Non-inflammatory Mediators*							
CGRP (amino acid)	CNS (predominant in DH of spinal cord) & PNS	CALCRL: AC/cAMP/PKA	CALCRL: CNS (nucleus accumbens), PNS (cardiovascular, immune, respiratory, endocrine, primary afferent neurons) (−/+)	CGRP	Excitatory	1. Synergistic with excitatory effect of SP	[55,56,57,58,59]
GABA (Amino acid)	CNS & PNS	GABA_A_: Cl^−^/K^+^ (IPSP) GABA_B_: AC/cAMP/PKA	GABA_A_: CNS, PNS (immune cells, endocrine tissues) GABA_B_: CNS, PNS (+/+)	GABA: muscimol, isoguvacine, gaboxadol, progabide	Inhibitory (GABA_A_ & GABA_B_)		[60,61]
Opioid Peptides (Neuropeptide)	CNS (hypothalamus, striatum, spinal cord, hippocampus) & PNS	KOR, MOR & DOR: AC/cAMP/PKA	KOR: CNS (PAG, RVM, brain, spinal cord), PNS (primary afferent neurons)MOR: CNS (PAG, RVM, cerebral cortex, amygdala, DH of spinal cord)DOR: CNS (basal ganglia, neocortical region) (+/+)	MOR: enkephalins & β-endorphins (high affinity) KOR: Dynorphins (high affinity) DOR: Enkephalins	Inhibitory		[62,63,64]
Glycine (Amino acid)	CNS	GlyR: Cl^−^ (IPSP)	CNS (−/+)	Glycine, β-alanine, Taurine	Inhibitory		[65,66]
Cannabinoids (Lipid)	CNS (brain) & PNS	CB_1_ & CB_2_: AC/cAMP/PKA	CB_1_: CNS (brain and DH of spinal cord), PNS (lungs, kidneys, liver) (+/+) CB_2_: CNS (brainstem), PNS (immune cells, hematopoietic cells) (+/−)	Cannabinoids: THC, Anandamide, 2-Arachidonoylglycerol, 2-Arachidonyl glyceryl ether, N-Arachidonoyl dopamine, Virodhamine	Inhibitory (CB_1_ & CB_2_)	1. Prevent the mast cells degranulation and the release of pro-inflammatory mediators	[67,68,69,70,71]

+: Presence; −: Absence; 5-HT: 5-hydroxytryptamine; 5-HT_2A_: 5-hydroxytryptamine type 2A receptor; 5-HT_3_: 5-hydroxytryptamine type 3 receptor; A_1_: adenosine type 1 receptor; A_2_: adenosine type 2 receptor; AA: arachidonic acid; AC: adenylyl cyclase; AMPA-R: amino-3-hydroxy-5-methyl-4-isoxazolepropionic acid receptors; ASIC: acid-sensing ion channels; ATP: adenosine triphosphate; B_1_: bradykinin receptor type B1; B_2_: bradykinin receptor type B2; BK: bradykinin; CALCRL: calcitonin receptor-like receptor; cAMP: cyclic adenosine monophosphate; CB_1_: cannabinoid type 1 receptors; CB_2_: cannabinoid type 2 receptors; cGMP: cyclic guanosine monophosphate; CGRP: calcitonin gene-related peptide; Cl^−^: chloride ion; CNS: central nervous system; DAG: diacylglycerol; DH: dorsal horn; DOR: δ-opioid receptors; DRG: dorsal root ganglion; EP: prostaglandin E2 receptor; EP_1_: prostaglandin E2 receptor type 1; EP_2_: prostaglandin E2 receptor type 2; EP_3_: prostaglandin E2 receptor type 3; EP_4_: prostaglandin E2 receptor type 4; EPSP: excitatory postsynaptic potentials; GABA: γ-aminobutyric acid; GABA_A_: γ-aminobutyric acid type A receptor; GABA_B_: γ-aminobutyric acid type B receptor; GI: gastrointestinal; GlyR: glycine receptor; H_1_: histamine; IL-2: interleukin-2; IP: prostacyclin receptor; IP_3_: inositol triphosphate; IPSP: inhibitory postsynaptic potentials; K^+^: potassium ion; KOR: κ-opioid receptors; LTB_4_: leukotriene B_4_; LTB_4_-R_1_: leukotriene B_4_ type 1 receptor; LTB_4_-R_2_: leukotriene B_4_ type 2 receptor; Mg^2+^: magnesium ion; MOR: µ-opioid receptors; Na^+^: sodium ion; Na_v_: voltage-activated Na^+^ channels; NE: norepinephrine; NGF: nerve growth factor; NK_1_: neurokinin type 1 receptor; NK_2_: neurokinin type 2 receptor; NK_3_: neurokinin type 3 receptor; NKA: neurokinin A; NKB: neurokinin B; NMDA-R: N-methyl-D-aspartate receptors; NO: nitric oxide; P2X_3_: purino receptor; PAG: periaqueductal gray; PG: prostaglandins; PGE_1_: prostaglandin E_1_; PGE_2_: prostaglandin E_2_; PGI_2_: prostacyclin; PI3: phosphoinositide 3-kinase; PKA: protein kinase A; PKC: protein kinase C; PLC: phospholipase C; PNS: peripheral nervous system; RVM: rostral ventral medulla; sGC: soluble guanylyl cyclase; SNS: sensory neuron specific; SP: substance P; THC: tetrahydrocannabinol; TrkA: tropomyosin receptor kinase A; VEC: vascular endothelial cell; VR1: vanilloid receptor for capsaicin; VSMC: vascular smooth muscle cell; α1: alpha 1-adrenoreceptor; α2: alpha 2-adrenoreceptor; β: beta-adrenoreceptor.

**Table 2:** The pain-mediated intracellular effectors and their signaling mechanism pathways.

Intracellular Effectors		
*G-protein-coupled receptors (Metabotropic)*		
PLC/IP_3_, DAG/PKC	G_q_α-protein-coupled receptors	Excitatory
Inhibit NE release, Inhibit AC/cAMP/PKA	G_i_α-protein-coupled receptors	Inhibitory
Activate AC/cAMP/PKA	G_s_α-protein-coupled receptors	Excitatory
sGC/cGMP	NO-signaling cascade	Inhibitory
*Ligand-gated ion channels (Ionotropic)*		
Cl^−^	GABA_A_	Inhibitory
Na^+^	SNS Na_v_	Excitatory
Ca^2+^	VOCC	Excitatory
K^+^	K_v_	Inhibitory
Protons (H^+^)	ASIC, VR1	Excitatory

Two important properties of receptors include (1) the recognition of extracellular molecules; and (2) transduction of the signals down the cascades for excitatory/inhibitory pharmacological response via (a) the ions movement across the membrane; (b) phosphorylation of protein kinases; (c) changes of transmitters released; (d) protein synthesis regulation; and (e) enzymatic activity. PKA and PKC can interact and sensitize SNS Na_v_ and VR1 receptors. AC: adenylyl cyclase; ASIC: acid-sensing ion channels; Ca^2+^: calcium ion; cAMP: cyclic adenosine monophosphate; cGMP: cyclic guanosine monophosphate; Cl^−^: chloride ion; DAG: diacylglycerol; GABA_A_: γ-aminobutyric acid type A; IP_3_: inositol triphosphate; K^+^: potassium ion; K_v_: voltage-activated potassium channels; Na^+^: sodium ion; Na_v_: voltage-activated Na^+^ channels; NE: norepinephrine; NO: nitric oxide; PKA: protein kinase A; PKC: protein kinase C; PLC: phospholipase C; sGC: soluble guanylyl cyclase; SNS: sensory neurone specific; VOCC: voltage-operated calcium channels; VR1: vanilloid receptor for capsaicin.
